# Usefulness of fluorescent ureteral catheter during laparoscopic residual ureterectomy

**DOI:** 10.1002/iju5.12680

**Published:** 2023-12-19

**Authors:** Tomoko Honda, Yuki Matsuoka, Yu Osaki, Yoichiro Tohi, Hirohito Naito, Takuma Kato, Homare Okazoe, Rikiya Taoka, Nobufumi Ueda, Mikio Sugimoto

**Affiliations:** ^1^ Department of Urology, Faculty of Medicine Kagawa University Kita‐gun Kagawa Japan

**Keywords:** fluorescent ureteral catheter, laparoscopic residual ureterectomy

## Abstract

**Introduction:**

There have been reports of surgery for residual ureteral tumors, most of them involved open surgeries. Herein, we report a case of retroperitoneal scopic left ureteral resection and partial cystectomy, performed by placing a fluorescent ureteral catheter in the residual ureter.

**Case presentation:**

A 79‐year‐old man was admitted to our hospital with a chief complaint of gross hematuria. He had undergone transperitoneal left radical nephrectomy due to angiomyolipoma 20 years ago. Computed tomography and Magnetic resonance imaging revealed a solid tumor in the left residual ureter. Retroperitoneal scopic residual ureterectomy has been performed. During the operation, a fluorescent ureteral catheter proved to be very helpful in detecting the ureter.

**Conclusion:**

A fluorescent ureteral catheter is considered to be a useful tool in laparoscopic surgery, especially in cases where identification of the ureter is expected to be difficult, such as the residual ureter in this case.

**Abbreviations & Acronyms**
CT: computed tomography
MRI: magnetic resonance imaging

**Keynote message**

There have been reports of surgery for residual ureteral tumors, most of them involved open surgeries. We report a case of retroperitoneal scopic left ureteral resection and partial cystectomy, performed by placing a fluorescent ureteral catheter in the residual ureter. A fluorescent ureteral catheter is considered to be a useful tool in laparoscopic surgery.

## Introduction

Primary ureteral tumors appearing in the residual ureter which were due to the history of prior nephrectomy are rare.^1^

We experienced a case of a patient who had a history of nephrectomy for angiomyolipoma 20 years prior and developed a tumor in the residual ureter. Although there have been reports of surgery for residual ureteral tumors, most of them involved open surgeries. Herein, we report a case of retroperitoneal scopic left ureteral resection and partial cystectomy, performed by placing a fluorescent ureteral catheter in the residual ureter. This is the first report of Laparoscopic surgery on the residual ureter.

## Case presentation

**Fig. 1:**
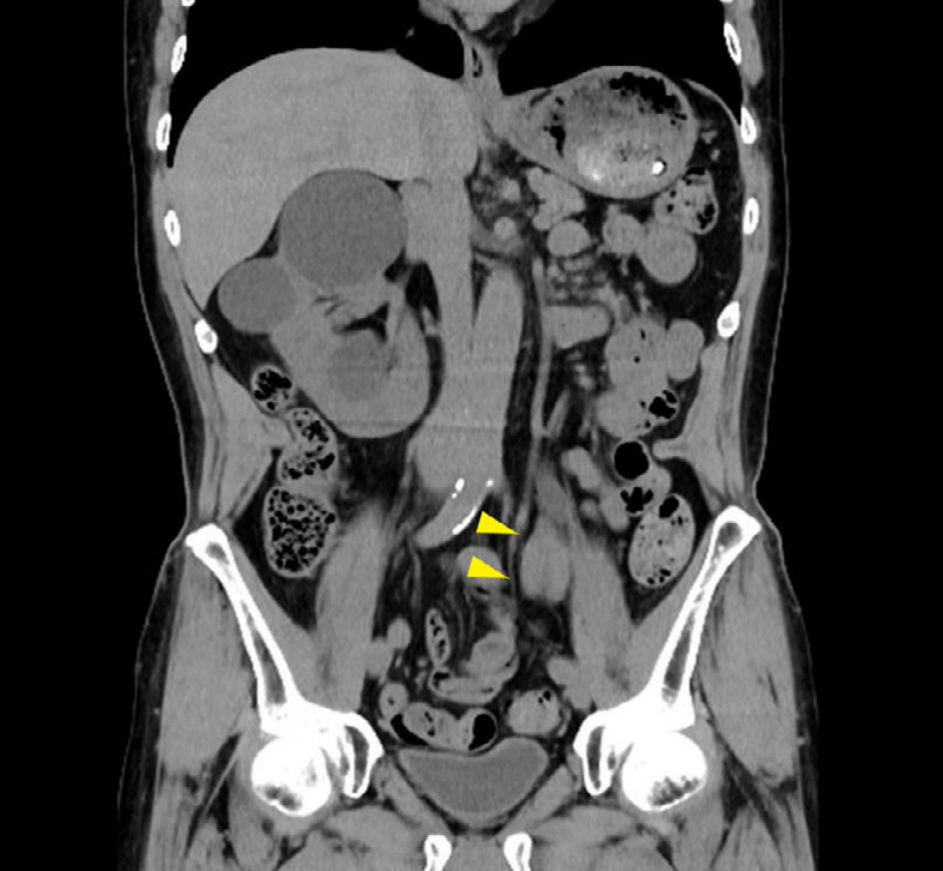
Computed tomography. A solid tumor measuring 41 × 23 mm was located on the left residual ureter (arrow).

A 79‐year‐old man, with a history of smoking (Brinkman Index = 75) and atrial fibrillation while taking rivaroxaban, visited the urologist because of gross hematuria. He had undergone a left transperitoneal open nephrectomy for angiomyolipoma 20 years ago, and there were no problems with the intraoperative and postoperative course. CT showed a soft tissue density tumor (41 × 23 mm) in the left lower ureter (Fig. 1). MRI revealed a tumor in the same area with a hyperintense signal on T2‐weighted images with diffusion restriction on the diffusion‐weighted image. Washing cytology of the left ureter indicated high‐grade urothelial carcinoma (class IV). Based on these findings, the patient was diagnosed with a ureteral tumor in the residual ureter and referred to our hospital for surgical treatment.

Owing to the expected difficulty in identifying the residual ureter during the operation, we decided to perform a retroperitoneoscopic left ureteral resection and partial cystectomy after placing a fluorescent ureteral catheter in the residual ureter.

### Operative intervention

Under general anesthesia, the patient was placed in the lithotripsy position, and a 6Fr NIRC™ (Near‐Infrared Ray Catheter) fluorescent ureteral catheter (Nippon Covidien, Ltd., Tokyo, Japan) was inserted into the left residual ureter using cystoscopy. As expected preoperatively, the catheter of about 18 cm, which was the length from the left ureteral orifice to the blind end of the ureter, was inserted into the residual ureter. The catheter was easily inserted and there were no complications regarding stent placement.

In the right lateral position, a camera port was placed one finger's breadth away from the tip of the 12th rib. After dilating the retroperitoneal space with a Preperitoneal dissecting balloon, 12 mm ports were placed at the anterior and posterior axillary lines, respectively.

**Fig. 2:**
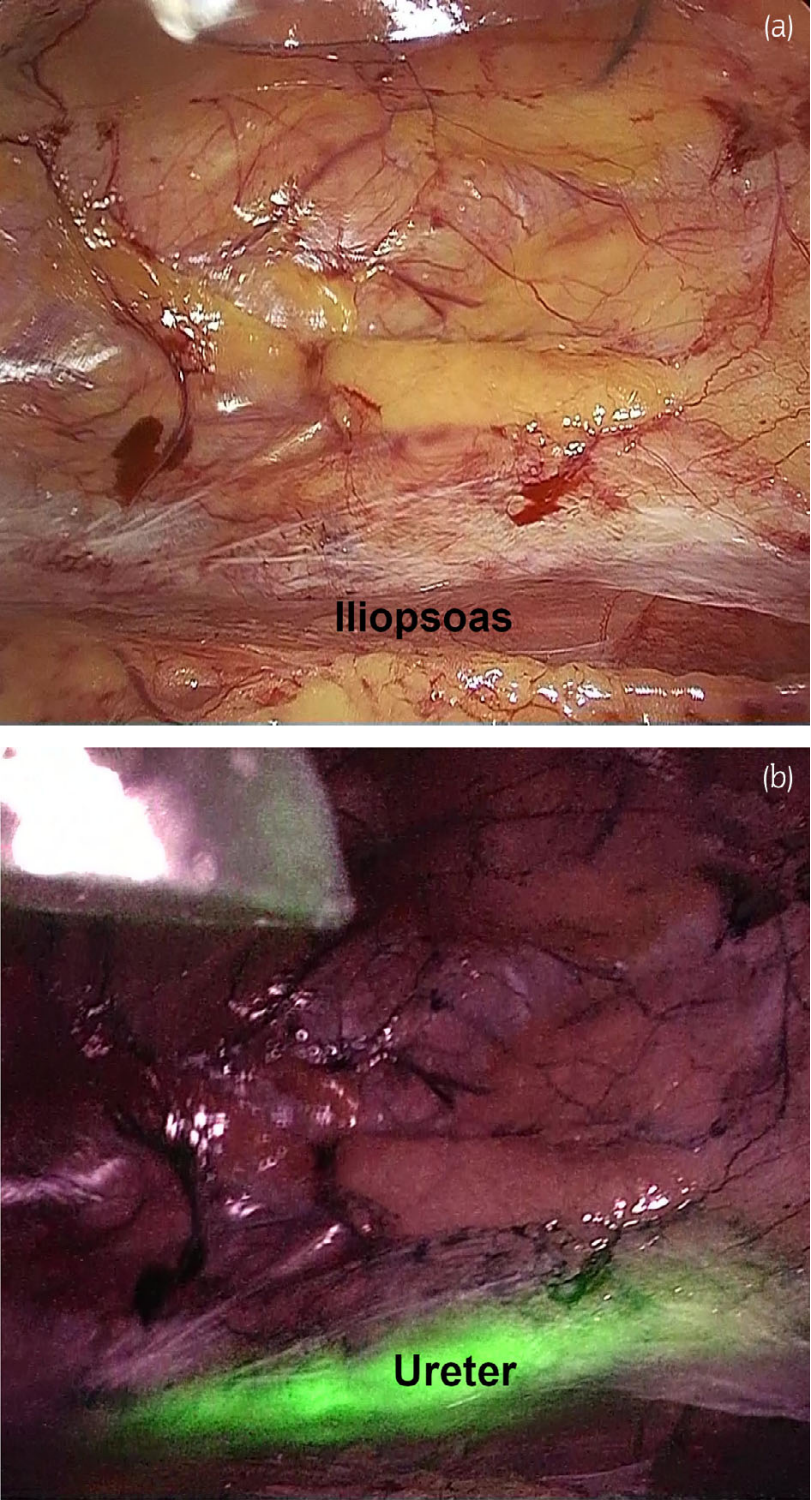
Intraoperative imaging. (a) Observation in normal mode. (b) Observation in near‐infrared mode. The ureter is visually recognized due to the green fluorescence emitted by the ureteral catheter.

Using near‐infrared light, we observed the ureteral catheter glowing green, enabling easy identification of the ureter (Fig. 2). The lateroconal fascia was incised at the level of the iliopsoas muscle. With the aid of near‐infrared light and visualization of the located ureter, we secured the ureter using vessel tape and dissected it to its intersection with the external iliac artery. The proximal stump of the ureter had severe adhesions as a result of a previous nephrectomy, making it difficult to identify the ureter without a fluorescent catheter. However, by performing the operation while visualizing the ureter using an ICG system (Olympus Co, Tokyo, Japan), it was possible to reliably and safely remove the ureter.

With the patient in the decubitus position, we proceeded with the lower ureteral resection. An 8‐cm pararectal incision was made to access the retroperitoneal space. The freed ureter was dissected from the bladder and removed along with the fluorescent ureteral catheter.

After separating the ureter from the bladder, we sutured the bladder mucosa and muscles into two layers. The operative time was 307 min, with the laparoscopic portion lasting 86 min and resulting in minimal bleeding.

**Fig. 3:**
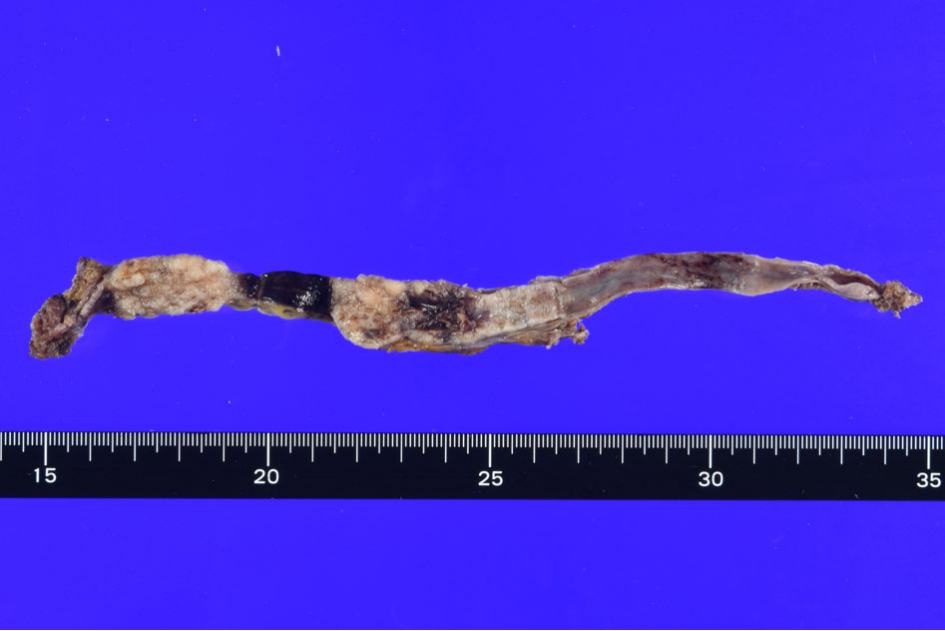
Excised specimen findings. Two white protruded lesions were observed in the lower ureter, measuring 5 and 3 cm in diameter, respectively.

The operative specimen revealed two protruding lesions in the lower ureter, measuring 5 and 3 cm in diameter, respectively (Fig. 3). The pathological diagnosis confirmed high‐grade invasive urothelial carcinoma (pT2).

### Postoperative course

The patient had an uneventful postoperative course. On postoperative day 8, cystography was performed, confirming no leaks. The ureteral catheter was subsequently removed, and the patient was discharged on postoperative day 9.

## Discussion

We reported a case in which laparoscopic surgery for a residual ureteral tumor was performed using a fluorescent ureteral catheter.

Ureteral tumors arising in the residual ureter after nephrectomy for benign disease are very rare.^1^ Malek *et al*. reported the occurrence of residual ureteral tumor in 4 out of 4883 patients (0.08%) who underwent nephrectomy for benign disease.^2^ Surgery for residual ureteral tumors has been reported, but most of them have been performed through a lower abdominal midline incision.^3^, ^4^ This may be attributed to the expected strong adhesion and difficulties in visualizing the ureter. In laparoscopic surgery, visualization plays an important role as palpation alone is challenging for identifying the ureter. Although conventional ureteral catheters have been used to locate the ureter, their effectiveness is limited. This is because conventional catheters may be difficult to visualize in cases with surrounding tissue adhesion, and the ureter cannot be recognized without touching the catheter‐inserted ureter.^5^ Recently, a near‐infrared ray catheter (NIRC™) fluorescent ureteral catheter (NIRFUC; Nippon Covidien, Ltd, Tokyo, Japan) has been developed, enabling visualization of the ureter under near‐infrared irradiation.^5^, ^6^ Fluorescent ureteral catheters have been reported to prevent ureteral injury, especially in gastrointestinal and gynecological surgeries.^5^, ^6^, ^7^

In this case, we considered the use of a fluorescent ureteral catheter a suitable indication due to the potential difficulties in visually identifying the ureter and the anticipated extensive adhesion resulting from the previous open nephrectomy. The point of this surgery was to check and keep the fluorescent ureteral catheter in position because the catheter might fall out easily during surgery. By using the fluorescent ureteral catheter, we successfully completed the operation laparoscopically, enabling minimally invasive surgery and smaller incisions.

## Conclusion

In conclusion, this report is the first case in which laparoscopic surgery using a fluorescent ureteral catheter was performed for a residual ureteral tumor, which is presumably the first report of its kind.

A fluorescent ureteral catheter is considered highly useful in laparoscopic surgery, particularly in cases where identification of the ureter is expected to be challenging.

## Author contributions

Tomoko Honda: Writing – original draft; writing – review and editing. Yuki Matsuoka: Writing – review and editing. Yu Osaki: Investigation. Yoichiro Tohi: Investigation. Hirohito Naito: Investigation. Takuma Kato: Supervision. Homare Okazoe: Supervision. Rikiya Taoka: Supervision. Nobufumi Ueda: Supervision. Mikio Sugimoto: Supervision.

## Conflict of interest

The authors declare no conflict of interest.

## Approval of the research protocol by an Institutional Reviewer Board

Not applicable.

## Informed consent

Informed consent was obtained from the patient for the publication of this case report and accompanying images.

## Registry and the Registration No. of the study/trial

Not applicable.
